# Crystal structures of the dimethyl sulfoxide solvate of 3,6-bis­(indol-3-yl)-1,4-di­methyl­piperazine-2,5-dione and of the dimethyl sulfoxide and tetra­hydro­furan solvates of 1,4-dimethyl-3,6-bis­(2-methyl­indol-3-yl)piperazine-2,5-dione

**DOI:** 10.1107/S205698902500920X

**Published:** 2025-10-28

**Authors:** Clifford W. Padgett, Will E. Lynch, Stephen N. Crooke, Christine R. Whitlock

**Affiliations:** a Department of Biochemistry Chemistry and Physics Georgia Southern University, Armstrong Campus 11935 Abercorn Street Savannah GA 31419 USA; b Center for Advanced Materials Science Department of Biochemistry Chemistry and Physics Georgia Southern University, 11935 Abercorn Street Savannah GA 31419 USA; c Department of Biochemistry Chemistry and Physics Georgia Southern University, Statesboro Campus 1332 Southern Drive Statesboro GA 30458 USA; University of Aberdeen, United Kingdom

**Keywords:** crystal structure, diketopiperazine, dimethyl sulfoxide (DMSO) solvate, tetra­hydro­furan (THF) solvate

## Abstract

The syntheses and structures of the dimethyl sulfoxide (DMSO) solvate of 3,6-bis­(indol-3-yl)-1,4-di­methyl­piperazine-2,5-dione, C_11_H_10_N_2_O (**I**), and of the dimethyl sulfoxide and tetra­hydro­furan (THF) solvates of 1,4-dimethyl-3,6-bis­(2-methyl­indol-3-yl)piperazine-2,5-dione, C_12_H_12_N_2_O, (**II**) and (**III**), respectively, are reported. The asymmetric units of (**I**) and (**II**) each contain two crystallographically independent half-mol­ecules that are completed by inversion symmetry, whereas (**III**) contains one independent half-mol­ecule. In all three structures, the piperazine-2,5-dione core is essentially planar and the overall mol­ecular non-planarity arises from rotations of the indole substituents: ranging between 58 and 63° in (**I**), approximately 72° for both independent mol­ecules in (**II**) and approximately 62° in (**III**). In the crystal of (**I**), mol­ecules are linked by two N—H⋯O hydrogen bonds to form *C*(18) chains; (**II**) features a single N—H⋯O contact giving *C*(8) chains; and (**III**) exhibits N—H⋯O inter­actions that generate *C*(7) chains assembling into sheets lying parallel to (100). No significant π–π stacking is present in any of these structures. All three structures contain regions of disordered solvent (DMSO or THF) that were treated with a solvent mask during refinement.

## Abstract 2

In the title solvates, the indole ring rotations (58–72°) relative to the plane of the central ring govern non-planarity. In the extended structures, N—H⋯O links form chains or sheets and disordered solvents were masked.

## 1. Chemical context

The bis­indolyl piperazine-2,5-dione motif has attracted considerable inter­est as a precursor to the dragmacidin family of marine natural products (Garg *et al.*, 2002 ▸). These alkaloids, isolated from deep-sea sponges and tunicates, are distinguished by a piperazine core bearing indole units at the 3- and 6-positions (Kawasaki *et al.*, 2002 ▸, 2003 ▸). Members of the dragmacidin series have been reported to display anti­cancer, anti­viral, anti-inflammatory and anti­bacterial activity (Cutignano *et al.*, 2000 ▸; Feldman & Ngernmeesri, 2011 ▸; Morris & Andersen, 1990 ▸; Wright *et al.*, 1992 ▸). Notably, dragmacidin, the first reported member, exhibits *in vitro* cytotoxic activity in A-549 (human lung), HCT-8 (human colon), P388 (murine leukemia) and MDA-MB (human mammary) cell lines (Kohmoto *et al.*, 1988 ▸).

3,6-Bis(indol-3-yl)-1,4-di­methyl­piperazine-2,5-dione and 1,4-dimethyl-3,6-bis­(2-methyl­indol-3-yl)piperazine-2,5-dione were prepared during efforts to access new dragmacidin derivatives (Crooke & Whitlock, 2012 ▸). In addition to a one-pot route, a reproducible two-step procedure was used: sarcosine anhydride was brominated, and the resulting precipitate was reacted with the appropriate indole in di­methyl­formamide (DMF) to afford the bis­indolyl products. As part of our work in this area, we now describe the syntheses and structures of the title compounds, each of which contain disordered solvent regions.
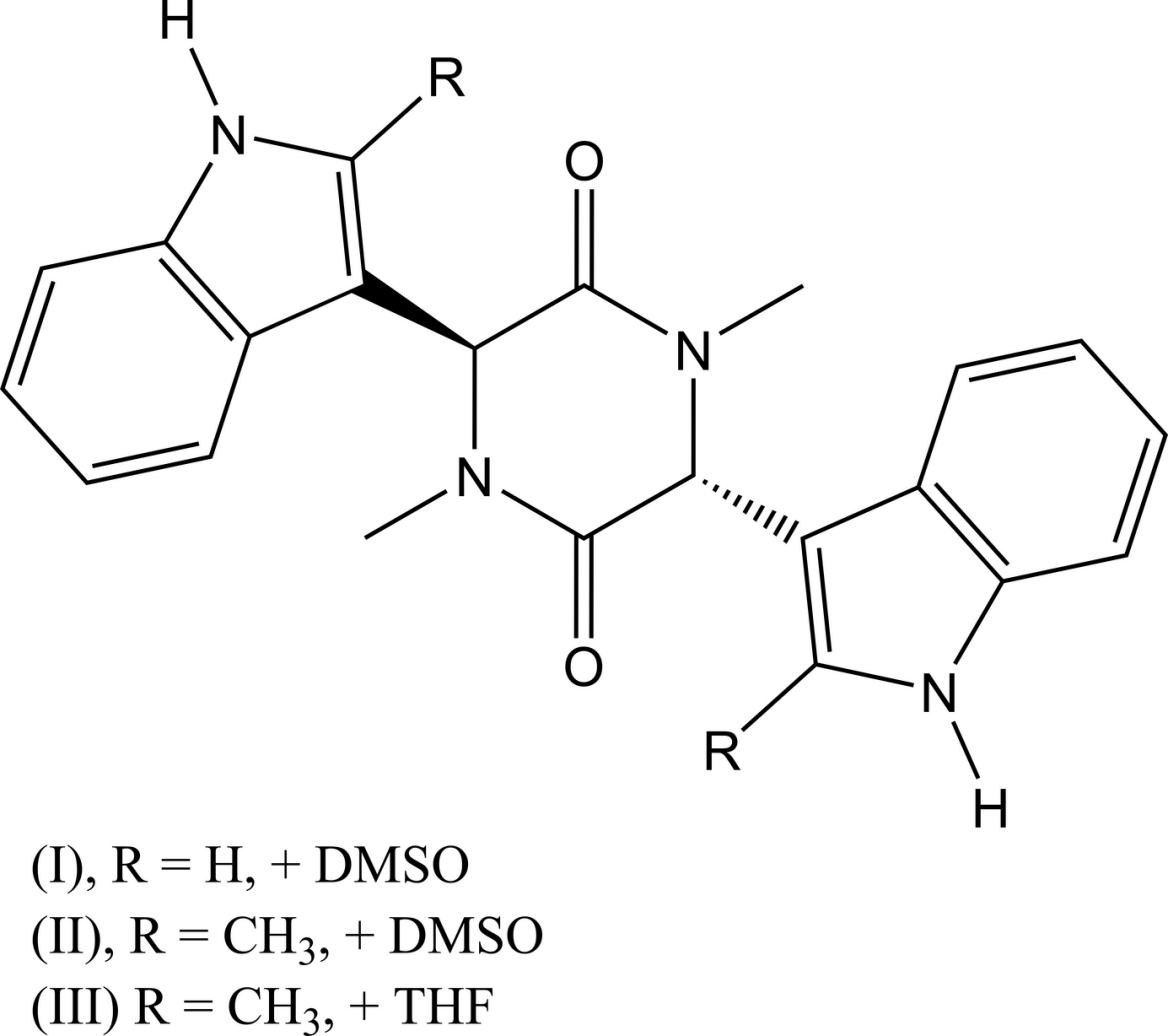


## 2. Structural commentary

**Figure 1:**
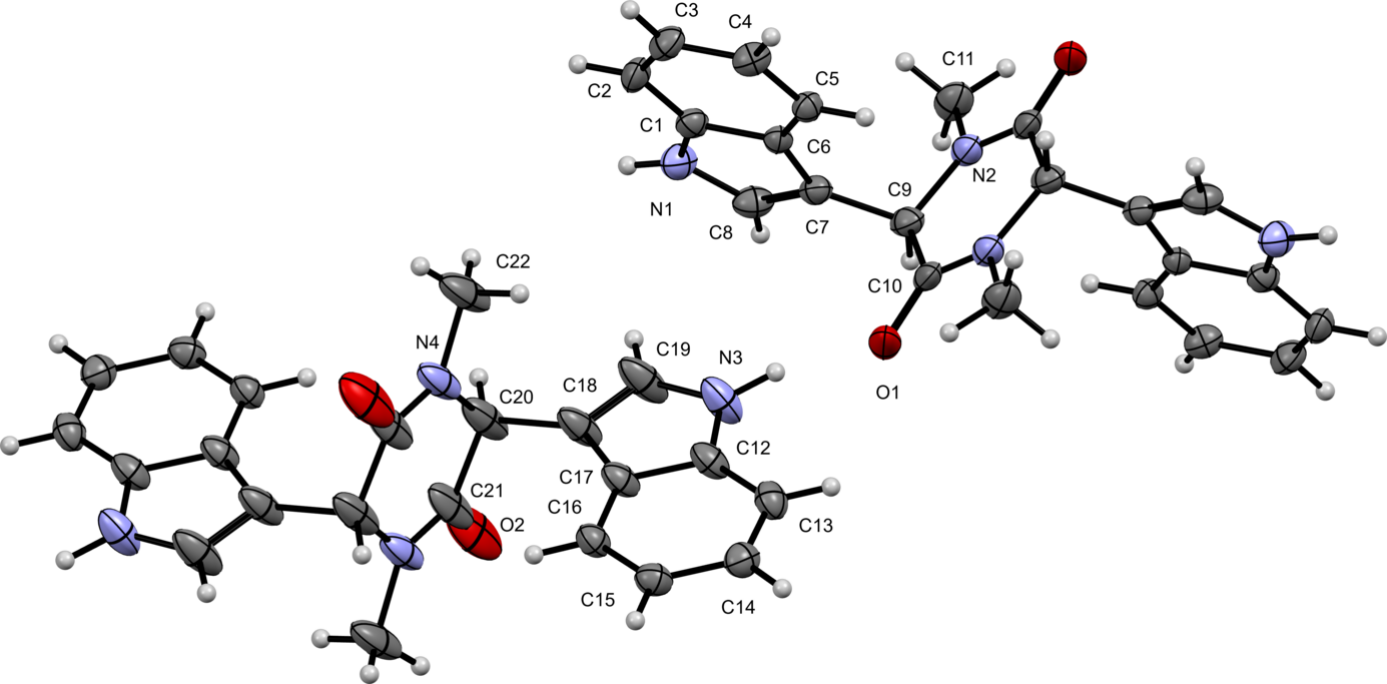
The mol­ecular structure of (**I**) with displacement ellipsoids drawn at the 50% probability level. The unlabelled atoms in the C1 mol­ecule are generated by the symmetry operation −*x* + 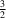, −*y* + 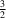, −*z* + 1 and those in the C12 mol­ecule by −*x* + 1, −*y*, −*z* + 1.

Compound (**I**) crystallizes in the monoclinic space group *C*2/c and its mol­ecular structure is shown in Fig. 1 ▸. The asymmetric unit contains two independent mol­ecular halves with each complete C_22_H_20_N_4_O_2_ mol­ecule generated by crystallographic inversion symmetry. Each indole ring shows an r.m.s. deviation of 0.004 Å. The indole ring containing N1 is rotated by 57.9 (2)° (C10—C9—C7—C6) relative to the piperazine-2,5-dione ring (r.m.s. deviation = 0.037 Å). Similarly, the indole ring containing N3 is rotated by 63.3 (3)° (C21—C20—C18—C17) relative to the piperazine-2,5-dione ring (r.m.s. deviation = 0.072 Å). These rotations are the principal contributors to the overall nonplanarity of the mol­ecule. The carbonyl C=O distances are normal at 1.234 (2) Å for C10=O1 and 1.230 (4) Å for C21=O2.

**Figure 2:**
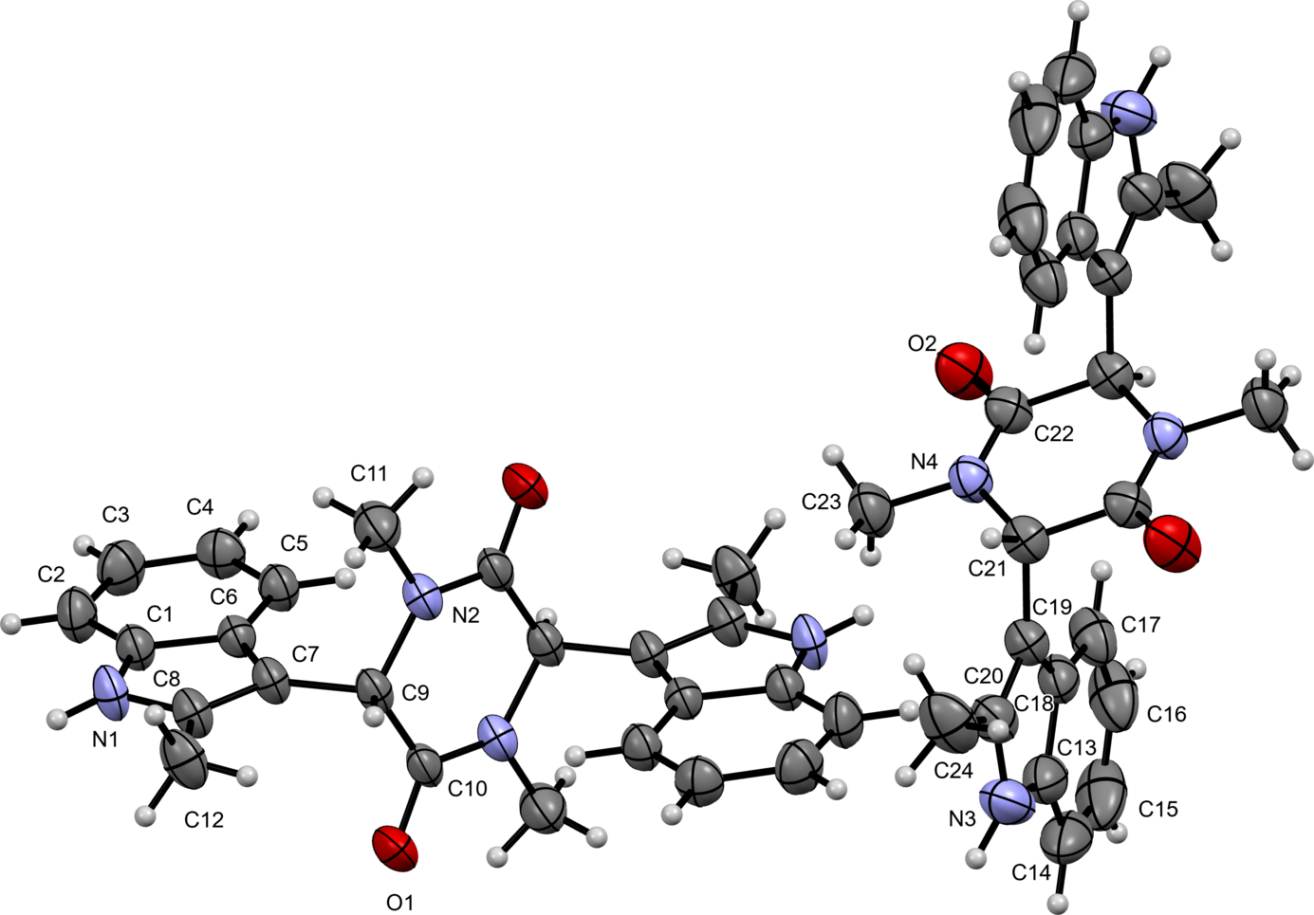
The mol­ecular structure of (**II**) with displacement ellipsoids drawn at the 50% probability level. The unlabelled atoms in the C1 mol­ecule are generated by the symmetry operation −*x* + 2, −*y* + 1, −*z* + 1 and those in the C13 mol­ecule by −*x*, −*y*, −*z*.

The mol­ecular structure of compound (**II**) (triclinic, space group *P*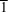) is shown in Fig. 2 ▸. The asymmetric unit also contains two independent mol­ecular halves completed by inversion symmetry to form C_24_H_24_N_4_O_2_ mol­ecules. The indole rings show r.m.s. deviations of 0.019 and 0.021 Å for the rings containing N1 and N3, respectively. The indole ring containing N1 is rotated by 71.4 (2)° (C10—C9—C7—C6) relative to the piperazine-2,5-dione ring (r.m.s. deviation = 0.037 Å). Similarly, the indole ring containing N3 is rotated by −72.7 (3)° (C22—C21—C19—C18) relative to the piperazine-2,5-dione ring (r.m.s. deviation = 0.046 Å). These rotations are the principal contributors to the overall nonplanarity of the mol­ecule. The carbonyl C=O distances are 1.232 (3) Å (C10=O1) and 1.226 (3) Å (C22=O2).

**Figure 3:**
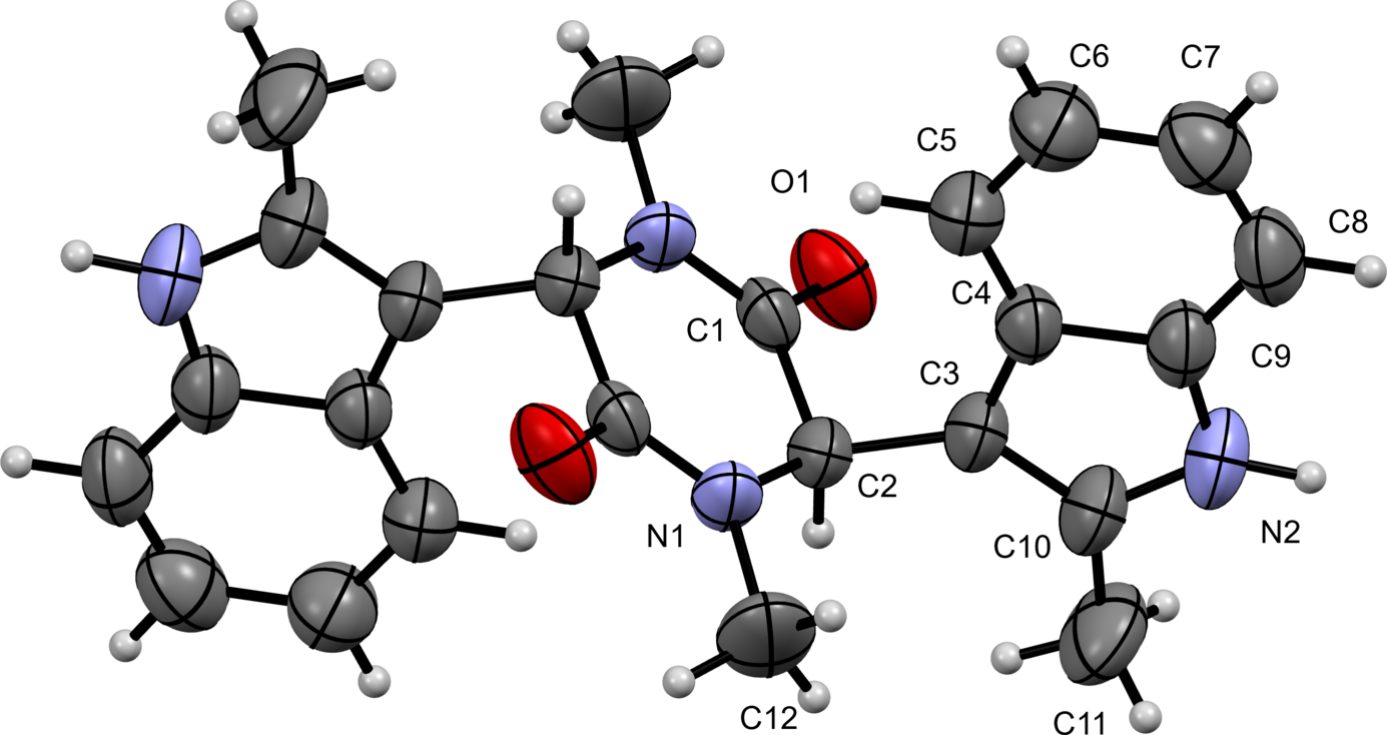
The mol­ecular structure of (**III**) with displacement ellipsoids drawn at the 50% probability level. The unlabelled atoms are generated by the symmetry operation –*x* + 1, –*y* + 1, –*z* + 1.

Compound (**III)** crystallizes in the monoclinic space group *P*2_1_/c and its mol­ecular structure is shown in Fig. 3 ▸. The asymmetric unit contains a half mol­ecule, which is completed by inversion symmetry to generate a C_24_H_24_N_4_O_2_ mol­ecule. The indole ring shows an r.m.s. deviation of 0.010 Å. The indole ring is rotated by −62.4 (3)° (C1—C2—C3—C4) relative to the piperazine-2,5-dione ring (r.m.s. deviation = 0.031 Å) and the carbonyl C1=O1 distance is 1.231 (2) Å.

All three structures contain disordered solvent regions that were masked (see *Refinement*).

## 3. Supra­molecular features

**Figure 4:**
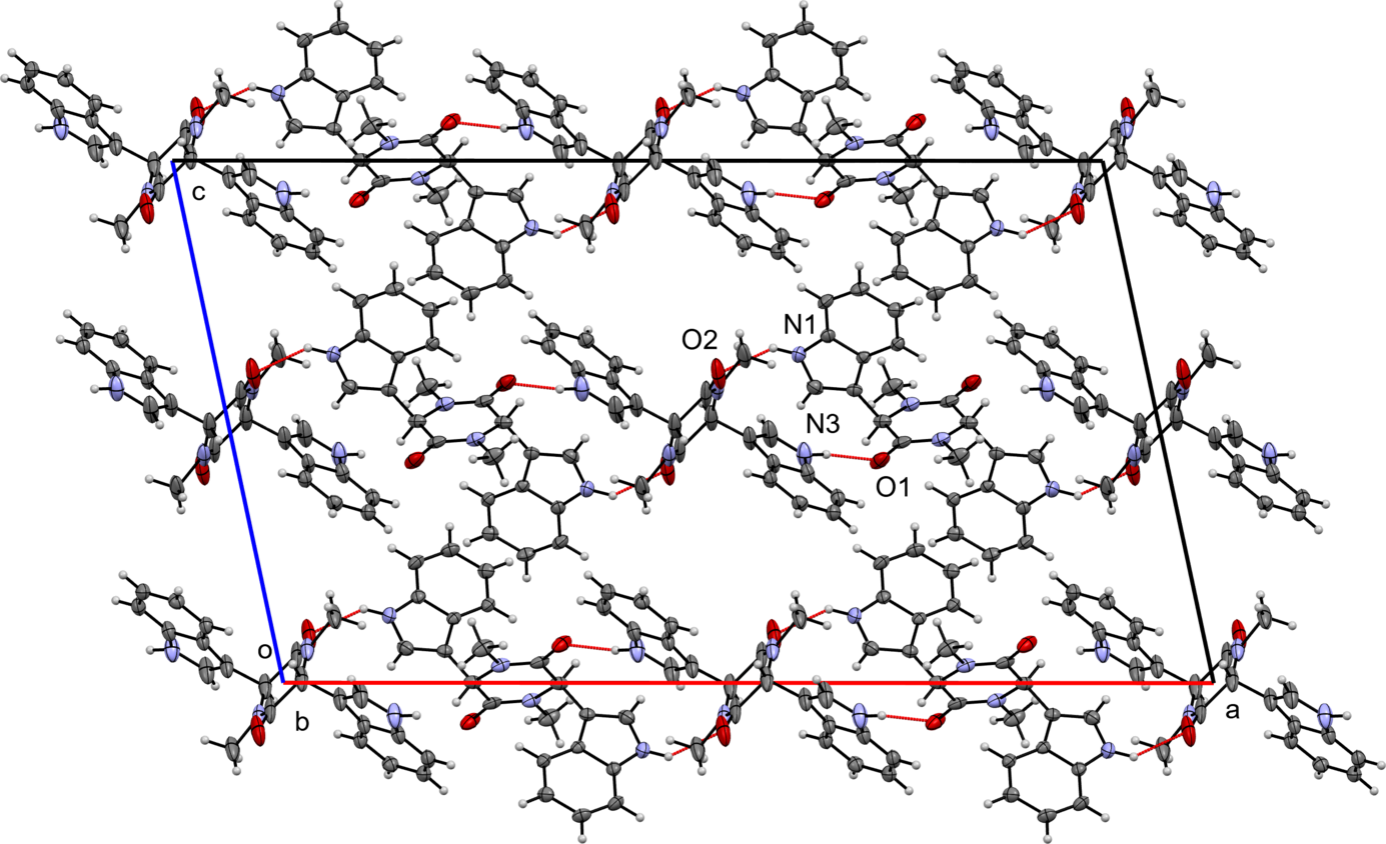
A view along the *b*-axis direction of the crystal packing of (**I**) with close contacts shown as red dashed lines.

In the crystal of (**I**), the mol­ecules are linked by two N—H⋯O hydrogen bonds (Table 1 ▸, Fig. 4 ▸): N1—H1⋯O2, which generates chains parallel to [1
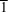
0] with graph-set motif 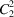
(18), and N3—H3⋯O1, forming chains along [130] with graph-set motif 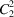
(18). Together, the hydrogen bonds generate (001) sheets. No significant aromatic π–π stacking is observed.

**Figure 5:**
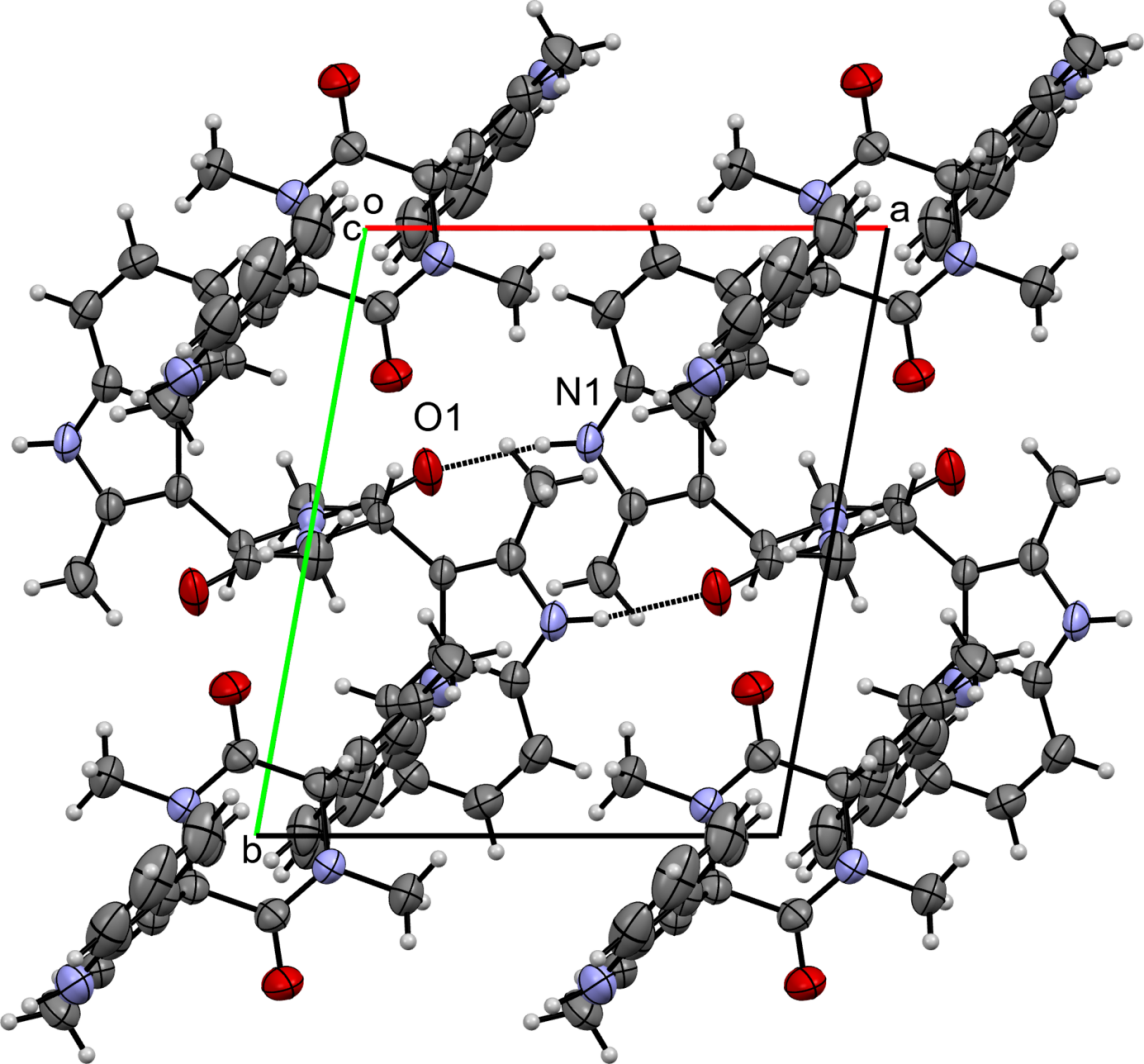
A view along the *c*-axis direction of the crystal packing of (**II**) with close contacts shown as red dashed lines.

In the crystal of (**II**), mol­ecules are linked by an N1—H1⋯O2 hydrogen bond (Table 2 ▸, Fig. 5 ▸), which generates chains running parallel to [100] with graph-set motif 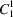
(8). The second mol­ecule (containing N3) is not involved in hydrogen bonding. No significant π–π stacking is observed.

**Figure 6:**
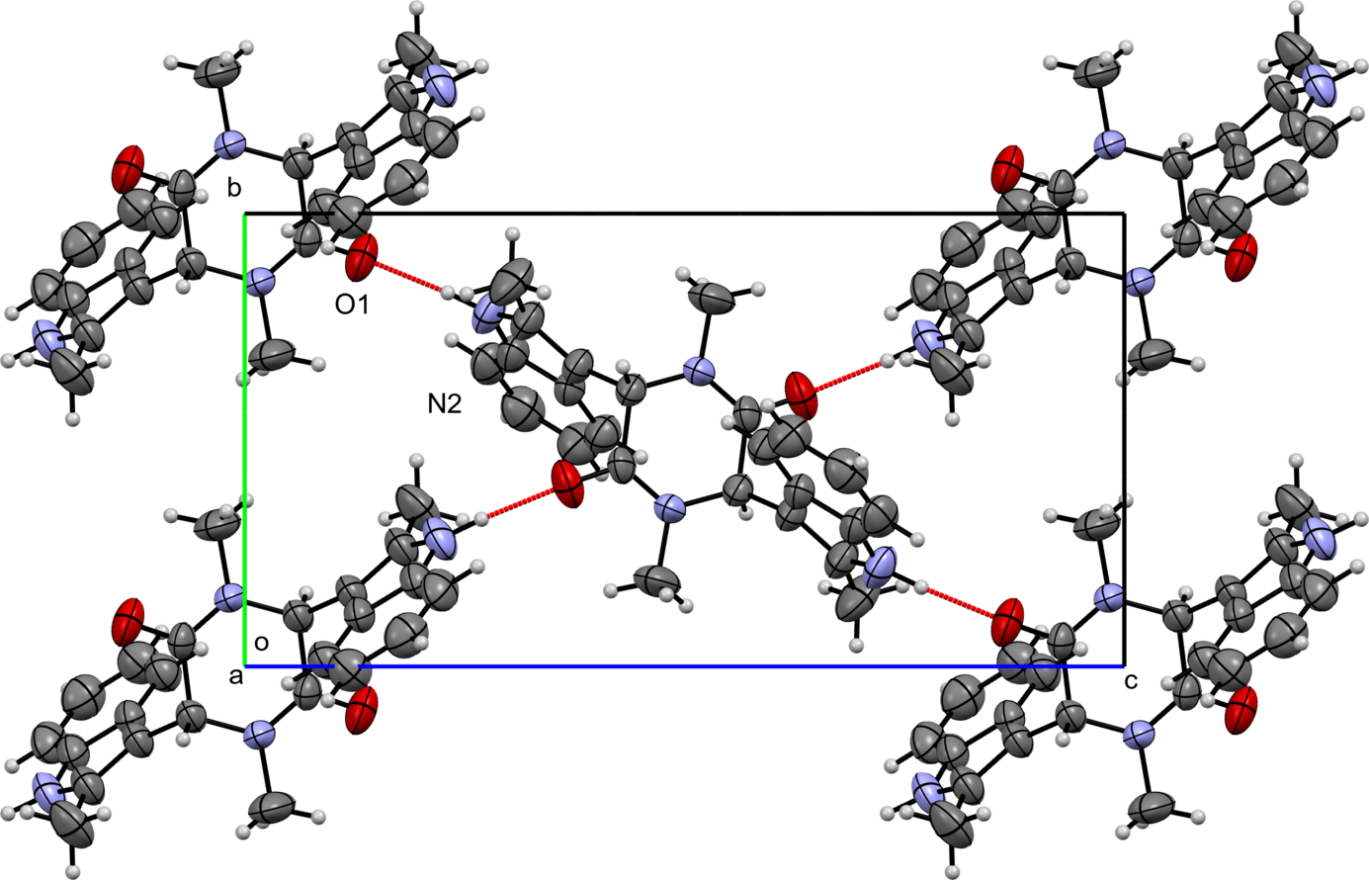
A view along the *a*-axis direction of the crystal packing of (**III**) with close contacts shown as red dashed lines.

In the crystal of (**III**), mol­ecules are linked by an N2—H2⋯O1 hydrogen bond (Table 3 ▸, Fig. 6 ▸), which generates chains of N—H⋯O hydrogen bonds [graph-set motif 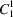
(7)] that form sheets parallel to the (100) plane. No significant π–π stacking is observed.

## 4. Database survey

A search of the Cambridge Structural Database ((CSD; website, accessed on August, 2025; Groom *et al.*, 2016 ▸) for 1,4-dimethyl-3,6-dioxopiperazine (diketopiperazine) frameworks bearing 2,6-substitution returned 13 structures. Seven entries feature simple alkyl groups; methyl (CSD refcode RMNALA10; Benedetti *et al.*, 1976 ▸) and isopropyl (NMLVAL10; Benedetti *et al.*, 1976 ▸), a mixed meth­yl/isopropyl pair (MOJTUB; Wang *et al.*, 2008 ▸), and 1,1,1-tri­fluoro­isopropyl (LAKGAF; Su *et al.*, 1993 ▸). Five entries carry benzylic/aromatic groups, including benzyl (NMLPHE11; Ge *et al.*, 2019 ▸) and (4-hy­droxy­phen­yl)methyl (NIPBIB; Croft *et al.*, 2004 ▸). One structure bears a carboxyl­ate protecting group, *tert*-but­oxy­carbonyl (EZESIO; Yang, 2021 ▸).

## 5. Synthesis and crystallization

To prepare compound (**I**), 0.0117 g of 3,6-bis­(indol-3-yl)-1,4-di­methyl­piperazine-2,5-dione (Miles & Whitlock, 2009 ▸) were dissolved in ∼10 ml of di­methyl­sulfoxide (DMSO) and heated to ∼423 K in a 50 ml beaker. The beaker was placed in a fume hood to allow slow evaporation for approximately 1 week, after which X-ray quality crystals began to form.

Compound (**II**): a 0.0079-g sample of 1,4-dimethyl-3,6-bis­(2-methyl­indol-3-yl)piperazine-2,5-dione (Miles & Whitlock, 2009 ▸) was dissolved in ∼5 ml of DMSO and heated to near boiling (∼423 K) in a 50 ml beaker. The beaker was placed in a fume hood to allow slow evaporation for approximately 1 week, after which X-ray quality crystals began to form.

Compound (**III**) was prepared by the same procedure as (**II**) except that 0.050 g of 1,4-dimethyl-3,6-bis­(2-methyl­indol-3-yl)piperazine-2,5-dione (0.05 g) (Miles & Whitlock, 2009 ▸) was dissolved in ∼50 ml of tetra­hydro­furan (THF) in a 100 ml beaker. The solution was allowed to slowly evaporate for approximately 1 week, after which X-ray quality crystals began to form.

## 6. Refinement

Crystal data, data collection and structure refinement details are summarized in Table 4 ▸. In all three structures, disordered solvent regions were treated using the built-in solvent-mask routine in *OLEX2*. Solvent-accessible voids were located in each unit cell with the following characteristics: for compound (**I**), a total cavity volume of 520 Å^3^ per unit cell containing 172 electrons (consistent with one C_2_H_6_OS mol­ecule per asymmetric unit, 168 electrons per cell); for compound (**II**), a 312 Å^3^ void with 85 electrons (one C_2_H_6_OS per asymmetric unit, 84 electrons per cell); and for compound (**III**), a 404 Å^3^ cavity holding 90 electrons (one C_4_H_8_O per asymmetric unit, 80 electrons per cell). All disordered solvent electron density was subsequently removed *via* the solvent-mask procedure.

## Supplementary Material

Crystal structure: contains datablock(s) I, II, III. DOI: 10.1107/S205698902500920X/hb8160sup1.cif

Structure factors: contains datablock(s) I. DOI: 10.1107/S205698902500920X/hb8160Isup2.hkl

Structure factors: contains datablock(s) II. DOI: 10.1107/S205698902500920X/hb8160IIsup3.hkl

Structure factors: contains datablock(s) III. DOI: 10.1107/S205698902500920X/hb8160IIIsup4.hkl

Supporting information file. DOI: 10.1107/S205698902500920X/hb8160Isup5.cml

Supporting information file. DOI: 10.1107/S205698902500920X/hb8160IIsup6.cml

Supporting information file. DOI: 10.1107/S205698902500920X/hb8160IIIsup7.cml

CCDC references: 2496708, 2496707, 2496706

Additional supporting information:  crystallographic information; 3D view; checkCIF report

## Figures and Tables

**Table 1:** Hydrogen-bond geometry (Å, °) for (**I**)

*D*—H⋯*A*	*D*—H	H⋯*A*	*D*⋯*A*	*D*—H⋯*A*
N1—H1⋯O2^i^	0.89 (3)	2.10 (3)	2.828 (2)	138 (2)
N3—H3*A*⋯O1	0.95 (3)	1.86 (3)	2.730 (2)	151 (3)

Symmetry code: (i) 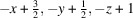.

**Table 2:** Hydrogen-bond geometry (Å, °) for (**II**)

*D*—H⋯*A*	*D*—H	H⋯*A*	*D*⋯*A*	*D*—H⋯*A*
N1—H1⋯O1^i^	0.85 (3)	2.11 (3)	2.950 (2)	168 (3)

Symmetry code: (i) 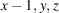.

**Table 3:** Hydrogen-bond geometry (Å, °) for (**III**)

*D*—H⋯*A*	*D*—H	H⋯*A*	*D*⋯*A*	*D*—H⋯*A*
N2—H2⋯O1^i^	0.91 (3)	1.89 (3)	2.787 (2)	168 (3)

Symmetry code: (i) 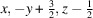.

**Table 4:** Experimental details

	(**I**)	(**II**)	(**III**)
Crystal data			
Chemical formula	C_22_H_20_N_4_O_2_·0.5C_2_H_6_OS	C_24_H_24_N_4_O_2_·C_2_H_6_SO	C_24_H_24_N_4_O_2_·C_4_H_8_O
*M*_r_	411.48	478.60	472.57
Crystal system, space group	Monoclinic, *C*2/*c*	Triclinic, *P* 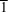	Monoclinic, *P*2_1_/*c*
Temperature (K)	100	298	293
*a*, *b*, *c* (Å)	31.2994 (4), 7.3881 (1), 17.9817 (2)	9.3137 (2), 12.3332 (2), 12.3873 (2)	8.5276 (9), 8.9357 (7), 17.4883 (13)
α, β, γ (°)	90, 102.023 (1), 90	117.110 (2), 93.520 (1), 97.399 (2)	90, 96.731 (8), 90
*V* (Å^3^)	4066.93 (9)	1244.35 (4)	1323.4 (2)
*Z*	8	2	2
Radiation type	Cu *K*α	Cu *K*α	Mo *K*α
μ (mm^−1^)	1.18	1.44	0.08
Crystal size (mm)	0.10 × 0.06 × 0.04	0.36 × 0.11 × 0.07	0.4 × 0.4 × 0.3

Data collection			
Diffractometer	XtaLAB Synergy, Single source at home/near, HyPix3000	XtaLAB Synergy, Single source at home/near, HyPix3000	XtaLAB Mini (ROW)
Absorption correction	Gaussian (*CrysAlis PRO*; Rigaku OD, 2023 ▸)	Multi-scan (*CrysAlis PRO*; Rigaku OD, 2023 ▸)	Multi-scan (*CrysAlis PRO*; Rigaku OD, 2023 ▸)
*T*_min_, *T*_max_	0.927, 1.000	0.436, 1.000	0.968, 1.000
No. of measured, independent and observed [*I* > 2σ(*I*)] reflections	12151, 3716, 3134	25135, 4560, 4029	6423, 2420, 1519
*R*_int_	0.027	0.039	0.031
(sin θ/λ)_max_ (Å^−1^)	0.602	0.602	0.602

Refinement			
*R*[*F*^2^ > 2σ(*F*^2^)], *wR*(*F*^2^), *S*	0.046, 0.120, 1.05	0.058, 0.177, 1.06	0.053, 0.178, 1.02
No. of reflections	3716	4560	2420
No. of parameters	263	283	143
H-atom treatment	H atoms treated by a mixture of independent and constrained refinement	H atoms treated by a mixture of independent and constrained refinement	H atoms treated by a mixture of independent and constrained refinement
Δρ_max_, Δρ_min_ (e Å^−3^)	0.21, −0.36	0.60, −0.21	0.26, −0.15

Computer programs: *CrysAlis PRO* (Rigaku OD, 2023 ▸), *SHELXT2018/2* (Sheldrick, 2015*a* ▸), *SHELXL2018/3* (Sheldrick, 2015*b* ▸) and *OLEX2* (Dolomanov *et al.*, 2009 ▸).

## supplementary crystallographic information

### 3,6-Bis(indol-3-yl)-1,4-dimethylpiperazine-2,5-dione dimethyl sulfoxide hemihydrate (I) Crystal data

**Table:**

C_22_H_20_N_4_O_2_·0.5C_2_H_6_OS	*F*(000) = 1736
*M**_r_* = 411.48	*D*_x_ = 1.344 Mg m^−^^3^
Monoclinic, *C*2/*c*	Cu *K*α radiation, λ = 1.54184 Å
*a* = 31.2994 (4) Å	Cell parameters from 6717 reflections
*b* = 7.3881 (1) Å	θ = 2.9–69.6°
*c* = 17.9817 (2) Å	µ = 1.18 mm^−^^1^
β = 102.023 (1)°	*T* = 100 K
*V* = 4066.93 (9) Å^3^	Block, clear colourless
*Z* = 8	0.10 × 0.06 × 0.04 mm

### 3,6-Bis(indol-3-yl)-1,4-dimethylpiperazine-2,5-dione dimethyl sulfoxide hemihydrate (I) Data collection

**Table:**

XtaLAB Synergy, Single source at home/near, HyPix3000 diffractometer	3716 independent reflections
Radiation source: micro-focus sealed X-ray tube, PhotonJet (Cu) X-ray Source	3134 reflections with *I* > 2σ(*I*)
Mirror monochromator	*R*_int_ = 0.027
Detector resolution: 10.0000 pixels mm^-1^	θ_max_ = 68.3°, θ_min_ = 2.9°
ω scans	*h* = −37→37
Absorption correction: gaussian (CrysAlisPro; Rigaku OD, 2023)	*k* = −8→8
*T*_min_ = 0.927, *T*_max_ = 1.000	*l* = −21→19
12151 measured reflections	

### 3,6-Bis(indol-3-yl)-1,4-dimethylpiperazine-2,5-dione dimethyl sulfoxide hemihydrate (I) Refinement

**Table:**

Refinement on *F*^2^	Primary atom site location: dual
Least-squares matrix: full	Hydrogen site location: mixed
*R*[*F*^2^ > 2σ(*F*^2^)] = 0.046	H atoms treated by a mixture of independent and constrained refinement
*wR*(*F*^2^) = 0.120	*w* = 1/[σ^2^(*F*_o_^2^) + (0.0451*P*)^2^ + 5.8441*P*] where *P* = (*F*_o_^2^ + 2*F*_c_^2^)/3
*S* = 1.05	(Δ/σ)_max_ = 0.001
3716 reflections	Δρ_max_ = 0.21 e Å^−^^3^
263 parameters	Δρ_min_ = −0.36 e Å^−^^3^
0 restraints	

### 3,6-Bis(indol-3-yl)-1,4-dimethylpiperazine-2,5-dione dimethyl sulfoxide hemihydrate (I) Special details

**Table:**

Geometry. All esds (except the esd in the dihedral angle between two l.s. planes) are estimated using the full covariance matrix. The cell esds are taken into account individually in the estimation of esds in distances, angles and torsion angles; correlations between esds in cell parameters are only used when they are defined by crystal symmetry. An approximate (isotropic) treatment of cell esds is used for estimating esds involving l.s. planes.

### 3,6-Bis(indol-3-yl)-1,4-dimethylpiperazine-2,5-dione dimethyl sulfoxide hemihydrate (I) Fractional atomic coordinates and isotropic or equivalent isotropic displacement parameters (Å^2^)

**Table:**

	*x*	*y*	*z*	*U*_iso_*/*U*_eq_	
O1	0.69198 (5)	0.5128 (2)	0.42587 (8)	0.0444 (4)	
N2	0.76249 (5)	0.5922 (2)	0.46638 (8)	0.0308 (4)	
N1	0.87085 (5)	0.8346 (3)	0.37106 (9)	0.0344 (4)	
O2	0.53832 (5)	−0.2656 (3)	0.59204 (13)	0.0703 (6)	
C6	0.80197 (6)	0.9116 (2)	0.37659 (9)	0.0220 (4)	
N3	0.61216 (6)	0.3788 (3)	0.43637 (13)	0.0474 (5)	
N4	0.53359 (5)	0.0338 (3)	0.56374 (12)	0.0489 (5)	
C1	0.83537 (6)	0.9333 (3)	0.33524 (10)	0.0264 (4)	
C5	0.76208 (6)	1.0008 (2)	0.35093 (9)	0.0250 (4)	
H5	0.739130	0.989127	0.377837	0.030*	
C7	0.81931 (6)	0.7946 (3)	0.43927 (9)	0.0258 (4)	
C9	0.79628 (6)	0.7240 (3)	0.49886 (10)	0.0296 (4)	
H9	0.818533	0.658316	0.537430	0.035*	
C10	0.71973 (7)	0.6176 (3)	0.46083 (10)	0.0314 (4)	
C4	0.75654 (6)	1.1058 (3)	0.28604 (10)	0.0299 (4)	
H4	0.729678	1.167259	0.268447	0.036*	
C16	0.56976 (6)	−0.0656 (3)	0.39021 (11)	0.0303 (4)	
H16	0.546853	−0.137952	0.401555	0.036*	
C8	0.86087 (6)	0.7530 (3)	0.43377 (10)	0.0329 (4)	
H8	0.880200	0.678246	0.468420	0.040*	
C3	0.79030 (7)	1.1224 (3)	0.24595 (10)	0.0325 (4)	
H3	0.785603	1.193938	0.201093	0.039*	
C17	0.57703 (6)	0.1098 (3)	0.42017 (12)	0.0334 (4)	
C15	0.59634 (6)	−0.1316 (3)	0.34402 (11)	0.0315 (4)	
H15	0.591717	−0.250734	0.324046	0.038*	
C2	0.82979 (6)	1.0385 (3)	0.26952 (10)	0.0315 (4)	
H2	0.852523	1.051494	0.242183	0.038*	
C14	0.63015 (6)	−0.0256 (3)	0.32604 (11)	0.0324 (4)	
H14	0.647679	−0.074018	0.293596	0.039*	
C12	0.61152 (6)	0.2127 (3)	0.40155 (12)	0.0346 (5)	
C13	0.63833 (6)	0.1467 (3)	0.35452 (11)	0.0338 (4)	
H13	0.661286	0.217965	0.342668	0.041*	
C11	0.77761 (8)	0.4289 (3)	0.43354 (12)	0.0408 (5)	
H11A	0.783541	0.457243	0.383422	0.061*	
H11B	0.755010	0.335199	0.428275	0.061*	
H11C	0.804409	0.384838	0.466972	0.061*	
C18	0.55721 (6)	0.2240 (3)	0.46845 (14)	0.0447 (6)	
C21	0.52059 (7)	−0.1388 (4)	0.55313 (16)	0.0543 (7)	
C20	0.52044 (7)	0.1779 (4)	0.50718 (15)	0.0515 (7)	
H20	0.514382	0.288344	0.535425	0.062*	
C19	0.57983 (7)	0.3831 (3)	0.47672 (16)	0.0540 (7)	
H19	0.573892	0.482684	0.506405	0.065*	
C22	0.56984 (7)	0.0801 (4)	0.62727 (16)	0.0614 (8)	
H22A	0.570409	−0.005583	0.669088	0.092*	
H22B	0.565694	0.203079	0.644882	0.092*	
H22C	0.597536	0.073426	0.610121	0.092*	
H1	0.8970 (9)	0.837 (4)	0.3587 (14)	0.054 (7)*	
H3A	0.6357 (10)	0.461 (4)	0.4377 (17)	0.076 (9)*	

### 3,6-Bis(indol-3-yl)-1,4-dimethylpiperazine-2,5-dione dimethyl sulfoxide hemihydrate (I) Atomic displacement parameters (Å^2^)

**Table:**

	*U*^11^	*U*^22^	*U*^33^	*U*^12^	*U*^13^	*U*^23^
O1	0.0531 (9)	0.0501 (9)	0.0362 (7)	−0.0279 (8)	0.0232 (7)	−0.0200 (7)
N2	0.0444 (10)	0.0248 (8)	0.0255 (8)	−0.0095 (7)	0.0124 (7)	−0.0039 (6)
N1	0.0252 (8)	0.0467 (10)	0.0321 (8)	0.0051 (8)	0.0080 (7)	−0.0045 (8)
O2	0.0217 (8)	0.0698 (13)	0.1167 (17)	0.0058 (9)	0.0079 (9)	−0.0323 (12)
C6	0.0252 (8)	0.0206 (8)	0.0209 (8)	−0.0030 (7)	0.0067 (6)	−0.0032 (7)
N3	0.0293 (9)	0.0349 (10)	0.0782 (14)	−0.0068 (8)	0.0118 (9)	−0.0178 (10)
N4	0.0218 (8)	0.0628 (14)	0.0636 (12)	−0.0095 (9)	0.0121 (8)	−0.0367 (11)
C1	0.0258 (9)	0.0288 (10)	0.0253 (9)	−0.0001 (8)	0.0065 (7)	−0.0057 (7)
C5	0.0263 (9)	0.0259 (9)	0.0243 (8)	−0.0006 (7)	0.0088 (7)	−0.0022 (7)
C7	0.0292 (9)	0.0256 (9)	0.0218 (8)	−0.0011 (8)	0.0037 (7)	−0.0022 (7)
C9	0.0392 (10)	0.0282 (10)	0.0203 (8)	−0.0052 (8)	0.0039 (7)	0.0005 (7)
C10	0.0474 (11)	0.0303 (10)	0.0194 (8)	−0.0143 (9)	0.0139 (8)	−0.0034 (7)
C4	0.0338 (10)	0.0279 (10)	0.0276 (9)	0.0063 (8)	0.0056 (7)	−0.0005 (8)
C16	0.0225 (9)	0.0314 (10)	0.0364 (10)	−0.0024 (8)	0.0052 (7)	−0.0047 (8)
C8	0.0328 (10)	0.0354 (11)	0.0282 (9)	0.0062 (9)	0.0006 (7)	−0.0025 (8)
C3	0.0440 (11)	0.0303 (10)	0.0250 (9)	0.0000 (9)	0.0108 (8)	0.0038 (8)
C17	0.0186 (8)	0.0348 (11)	0.0453 (11)	−0.0003 (8)	0.0031 (8)	−0.0109 (9)
C15	0.0301 (9)	0.0308 (10)	0.0325 (10)	0.0011 (8)	0.0042 (8)	−0.0048 (8)
C2	0.0358 (10)	0.0354 (11)	0.0275 (9)	−0.0046 (9)	0.0164 (8)	−0.0011 (8)
C14	0.0265 (9)	0.0398 (11)	0.0309 (9)	0.0049 (8)	0.0058 (7)	−0.0010 (9)
C12	0.0220 (9)	0.0316 (11)	0.0478 (11)	−0.0011 (8)	0.0015 (8)	−0.0056 (9)
C13	0.0230 (9)	0.0381 (11)	0.0397 (10)	−0.0015 (8)	0.0048 (8)	0.0036 (9)
C11	0.0601 (14)	0.0277 (11)	0.0393 (11)	−0.0066 (10)	0.0208 (10)	−0.0081 (9)
C18	0.0201 (9)	0.0458 (13)	0.0677 (15)	−0.0031 (9)	0.0082 (9)	−0.0293 (12)
C21	0.0170 (10)	0.0634 (17)	0.0854 (18)	−0.0041 (11)	0.0175 (11)	−0.0408 (15)
C20	0.0218 (10)	0.0555 (15)	0.0787 (17)	−0.0048 (10)	0.0141 (10)	−0.0425 (14)
C19	0.0269 (10)	0.0444 (14)	0.0899 (19)	−0.0028 (10)	0.0104 (11)	−0.0347 (13)
C22	0.0233 (10)	0.081 (2)	0.0773 (17)	−0.0086 (12)	0.0056 (11)	−0.0446 (16)

### 3,6-Bis(indol-3-yl)-1,4-dimethylpiperazine-2,5-dione dimethyl sulfoxide hemihydrate (I) Geometric parameters (Å, º)

**Table:**

O1—C10	1.234 (2)	C16—H16	0.9500
N2—C9	1.466 (2)	C16—C17	1.404 (3)
N2—C10	1.335 (3)	C16—C15	1.381 (3)
N2—C11	1.465 (3)	C8—H8	0.9500
N1—C1	1.372 (2)	C3—H3	0.9500
N1—C8	1.371 (3)	C3—C2	1.369 (3)
N1—H1	0.89 (3)	C17—C12	1.417 (3)
O2—C21	1.230 (4)	C17—C18	1.440 (3)
C6—C1	1.413 (2)	C15—H15	0.9500
C6—C5	1.402 (2)	C15—C14	1.407 (3)
C6—C7	1.434 (2)	C2—H2	0.9500
N3—C12	1.376 (3)	C14—H14	0.9500
N3—C19	1.363 (3)	C14—C13	1.377 (3)
N3—H3A	0.95 (3)	C12—C13	1.397 (3)
N4—C21	1.340 (3)	C13—H13	0.9500
N4—C20	1.471 (4)	C11—H11A	0.9800
N4—C22	1.473 (3)	C11—H11B	0.9800
C1—C2	1.395 (3)	C11—H11C	0.9800
C5—H5	0.9500	C18—C20	1.503 (3)
C5—C4	1.382 (3)	C18—C19	1.364 (3)
C7—C9	1.504 (2)	C21—C20^ii^	1.526 (3)
C7—C8	1.360 (3)	C20—H20	1.0000
C9—H9	1.0000	C19—H19	0.9500
C9—C10^i^	1.516 (3)	C22—H22A	0.9800
C4—H4	0.9500	C22—H22B	0.9800
C4—C3	1.403 (3)	C22—H22C	0.9800
			
C10—N2—C9	124.44 (17)	C16—C17—C12	118.38 (18)
C10—N2—C11	119.27 (17)	C16—C17—C18	135.76 (19)
C11—N2—C9	116.15 (17)	C12—C17—C18	105.86 (18)
C1—N1—H1	124.6 (17)	C16—C15—H15	119.4
C8—N1—C1	108.59 (16)	C16—C15—C14	121.16 (19)
C8—N1—H1	126.3 (17)	C14—C15—H15	119.4
C1—C6—C7	106.35 (15)	C1—C2—H2	121.3
C5—C6—C1	118.65 (16)	C3—C2—C1	117.39 (17)
C5—C6—C7	135.01 (16)	C3—C2—H2	121.3
C12—N3—H3A	121.3 (19)	C15—C14—H14	119.3
C19—N3—C12	108.71 (19)	C13—C14—C15	121.33 (18)
C19—N3—H3A	129.0 (19)	C13—C14—H14	119.3
C21—N4—C20	123.9 (2)	N3—C12—C17	108.09 (18)
C21—N4—C22	119.5 (3)	N3—C12—C13	129.3 (2)
C20—N4—C22	115.3 (2)	C13—C12—C17	122.63 (19)
N1—C1—C6	107.95 (16)	C14—C13—C12	117.34 (18)
N1—C1—C2	129.81 (17)	C14—C13—H13	121.3
C2—C1—C6	122.24 (17)	C12—C13—H13	121.3
C6—C5—H5	120.4	N2—C11—H11A	109.5
C4—C5—C6	119.23 (16)	N2—C11—H11B	109.5
C4—C5—H5	120.4	N2—C11—H11C	109.5
C6—C7—C9	127.58 (16)	H11A—C11—H11B	109.5
C8—C7—C6	106.87 (16)	H11A—C11—H11C	109.5
C8—C7—C9	125.51 (17)	H11B—C11—H11C	109.5
N2—C9—C7	111.08 (14)	C17—C18—C20	127.9 (2)
N2—C9—H9	107.1	C19—C18—C17	106.9 (2)
N2—C9—C10^i^	114.85 (16)	C19—C18—C20	125.1 (2)
C7—C9—H9	107.1	O2—C21—N4	123.8 (2)
C7—C9—C10^i^	109.13 (15)	O2—C21—C20^ii^	118.3 (2)
C10^i^—C9—H9	107.1	N4—C21—C20^ii^	117.8 (3)
O1—C10—N2	122.59 (19)	N4—C20—C18	110.70 (18)
O1—C10—C9^i^	117.43 (18)	N4—C20—C21^ii^	115.3 (2)
N2—C10—C9^i^	119.93 (17)	N4—C20—H20	107.1
C5—C4—H4	119.8	C18—C20—C21^ii^	109.0 (2)
C5—C4—C3	120.49 (17)	C18—C20—H20	107.1
C3—C4—H4	119.8	C21^ii^—C20—H20	107.1
C17—C16—H16	120.4	N3—C19—C18	110.4 (2)
C15—C16—H16	120.4	N3—C19—H19	124.8
C15—C16—C17	119.16 (18)	C18—C19—H19	124.8
N1—C8—H8	124.9	N4—C22—H22A	109.5
C7—C8—N1	110.24 (17)	N4—C22—H22B	109.5
C7—C8—H8	124.9	N4—C22—H22C	109.5
C4—C3—H3	119.0	H22A—C22—H22B	109.5
C2—C3—C4	121.99 (17)	H22A—C22—H22C	109.5
C2—C3—H3	119.0	H22B—C22—H22C	109.5
			
N1—C1—C2—C3	179.52 (19)	C8—C7—C9—N2	−107.9 (2)
C6—C1—C2—C3	0.2 (3)	C8—C7—C9—C10^i^	124.5 (2)
C6—C5—C4—C3	0.4 (3)	C17—C16—C15—C14	−0.6 (3)
C6—C7—C9—N2	69.8 (2)	C17—C12—C13—C14	−0.1 (3)
C6—C7—C9—C10^i^	−57.9 (2)	C17—C18—C20—N4	64.5 (3)
C6—C7—C8—N1	−0.7 (2)	C17—C18—C20—C21^ii^	−63.3 (3)
N3—C12—C13—C14	−179.1 (2)	C17—C18—C19—N3	0.8 (3)
C1—N1—C8—C7	0.7 (2)	C15—C16—C17—C12	0.1 (3)
C1—C6—C5—C4	0.4 (3)	C15—C16—C17—C18	179.7 (2)
C1—C6—C7—C9	−177.64 (17)	C15—C14—C13—C12	−0.4 (3)
C1—C6—C7—C8	0.4 (2)	C12—N3—C19—C18	−1.0 (3)
C5—C6—C1—N1	179.87 (16)	C12—C17—C18—C20	−176.9 (2)
C5—C6—C1—C2	−0.7 (3)	C12—C17—C18—C19	−0.3 (3)
C5—C6—C7—C9	2.6 (3)	C11—N2—C9—C7	61.3 (2)
C5—C6—C7—C8	−179.4 (2)	C11—N2—C9—C10^i^	−174.29 (16)
C5—C4—C3—C2	−0.9 (3)	C11—N2—C10—O1	−3.5 (3)
C7—C6—C1—N1	0.1 (2)	C11—N2—C10—C9^i^	173.93 (16)
C7—C6—C1—C2	179.48 (17)	C18—C17—C12—N3	−0.2 (2)
C7—C6—C5—C4	−179.87 (19)	C18—C17—C12—C13	−179.45 (19)
C9—N2—C10—O1	171.94 (17)	C21—N4—C20—C18	−104.3 (2)
C9—N2—C10—C9^i^	−10.6 (3)	C21—N4—C20—C21^ii^	20.0 (3)
C9—C7—C8—N1	177.38 (17)	C20—N4—C21—O2	163.8 (2)
C10—N2—C9—C7	−114.30 (19)	C20—N4—C21—C20^ii^	−20.5 (3)
C10—N2—C9—C10^i^	10.1 (3)	C20—C18—C19—N3	177.5 (2)
C4—C3—C2—C1	0.6 (3)	C19—N3—C12—C17	0.7 (3)
C16—C17—C12—N3	179.44 (18)	C19—N3—C12—C13	179.9 (2)
C16—C17—C12—C13	0.2 (3)	C19—C18—C20—N4	−111.5 (3)
C16—C17—C18—C20	3.5 (4)	C19—C18—C20—C21^ii^	120.6 (3)
C16—C17—C18—C19	−179.9 (2)	C22—N4—C21—O2	−2.9 (3)
C16—C15—C14—C13	0.8 (3)	C22—N4—C21—C20^ii^	172.8 (2)
C8—N1—C1—C6	−0.5 (2)	C22—N4—C20—C18	62.9 (2)
C8—N1—C1—C2	−179.8 (2)	C22—N4—C20—C21^ii^	−172.75 (18)

Symmetry codes: (i) −*x*+3/2, −*y*+3/2, −*z*+1; (ii) −*x*+1, −*y*, −*z*+1.

### 3,6-Bis(indol-3-yl)-1,4-dimethylpiperazine-2,5-dione dimethyl sulfoxide hemihydrate (I) Hydrogen-bond geometry (Å, º)

**Table:**

*D*—H···*A*	*D*—H	H···*A*	*D*···*A*	*D*—H···*A*
N1—H1···O2^iii^	0.89 (3)	2.10 (3)	2.828 (2)	138 (2)
N3—H3*A*···O1	0.95 (3)	1.86 (3)	2.730 (2)	151 (3)

Symmetry code: (iii) −*x*+3/2, −*y*+1/2, −*z*+1.

### 1,4-Dimethyl-3,6-bis(2-methylindol-3-yl)piperazine-2,5-dione dimethyl sulfoxide monosolvate (II) Crystal data

**Table:**

C_24_H_24_N_4_O_2_·C_2_H_6_SO	*Z* = 2
*M**_r_* = 478.60	*F*(000) = 508
Triclinic, *P*1	*D*_x_ = 1.277 Mg m^−^^3^
*a* = 9.3137 (2) Å	Cu *K*α radiation, λ = 1.54184 Å
*b* = 12.3332 (2) Å	Cell parameters from 17902 reflections
*c* = 12.3873 (2) Å	θ = 4.0–69.7°
α = 117.110 (2)°	µ = 1.44 mm^−^^1^
β = 93.520 (1)°	*T* = 298 K
γ = 97.399 (2)°	Block, clear colourless
*V* = 1244.35 (4) Å^3^	0.36 × 0.11 × 0.07 mm

### 1,4-Dimethyl-3,6-bis(2-methylindol-3-yl)piperazine-2,5-dione dimethyl sulfoxide monosolvate (II) Data collection

**Table:**

XtaLAB Synergy, Single source at home/near, HyPix3000 diffractometer	4560 independent reflections
Radiation source: micro-focus sealed X-ray tube, PhotonJet (Cu) X-ray Source	4029 reflections with *I* > 2σ(*I*)
Mirror monochromator	*R*_int_ = 0.039
Detector resolution: 10.0000 pixels mm^-1^	θ_max_ = 68.2°, θ_min_ = 4.1°
ω scans	*h* = −11→11
Absorption correction: multi-scan (CrysAlisPro; Rigaku OD, 2023)	*k* = −14→14
*T*_min_ = 0.436, *T*_max_ = 1.000	*l* = −14→14
25135 measured reflections	

### 1,4-Dimethyl-3,6-bis(2-methylindol-3-yl)piperazine-2,5-dione dimethyl sulfoxide monosolvate (II) Refinement

**Table:**

Refinement on *F*^2^	Primary atom site location: dual
Least-squares matrix: full	Hydrogen site location: mixed
*R*[*F*^2^ > 2σ(*F*^2^)] = 0.058	H atoms treated by a mixture of independent and constrained refinement
*wR*(*F*^2^) = 0.177	*w* = 1/[σ^2^(*F*_o_^2^) + (0.0886*P*)^2^ + 0.6913*P*] where *P* = (*F*_o_^2^ + 2*F*_c_^2^)/3
*S* = 1.06	(Δ/σ)_max_ < 0.001
4560 reflections	Δρ_max_ = 0.60 e Å^−^^3^
283 parameters	Δρ_min_ = −0.21 e Å^−^^3^
0 restraints	

### 1,4-Dimethyl-3,6-bis(2-methylindol-3-yl)piperazine-2,5-dione dimethyl sulfoxide monosolvate (II) Special details

**Table:**

Geometry. All esds (except the esd in the dihedral angle between two l.s. planes) are estimated using the full covariance matrix. The cell esds are taken into account individually in the estimation of esds in distances, angles and torsion angles; correlations between esds in cell parameters are only used when they are defined by crystal symmetry. An approximate (isotropic) treatment of cell esds is used for estimating esds involving l.s. planes.

### 1,4-Dimethyl-3,6-bis(2-methylindol-3-yl)piperazine-2,5-dione dimethyl sulfoxide monosolvate (II) Fractional atomic coordinates and isotropic or equivalent isotropic displacement parameters (Å^2^)

**Table:**

	*x*	*y*	*z*	*U*_iso_*/*U*_eq_	
O1	1.20329 (17)	0.40135 (17)	0.56994 (18)	0.0588 (5)	
O2	0.1008 (2)	0.24272 (15)	0.12756 (17)	0.0649 (5)	
N2	0.99719 (17)	0.48117 (16)	0.60324 (16)	0.0397 (4)	
N4	0.14924 (19)	0.05031 (16)	0.02277 (16)	0.0428 (4)	
N1	0.50383 (19)	0.35296 (18)	0.53767 (18)	0.0472 (5)	
C6	0.7058 (2)	0.30236 (18)	0.44973 (18)	0.0358 (4)	
C10	1.1103 (2)	0.44957 (18)	0.5413 (2)	0.0398 (5)	
C7	0.7330 (2)	0.43181 (18)	0.53668 (18)	0.0372 (4)	
N3	0.2971 (3)	−0.2443 (2)	0.1043 (2)	0.0638 (6)	
C9	0.87168 (19)	0.52304 (18)	0.56525 (19)	0.0373 (4)	
H9	0.859990	0.600449	0.635377	0.045*	
C1	0.5593 (2)	0.2567 (2)	0.4515 (2)	0.0411 (5)	
C8	0.6080 (2)	0.4585 (2)	0.58912 (19)	0.0416 (5)	
C5	0.7883 (2)	0.21910 (19)	0.37191 (19)	0.0419 (5)	
H5	0.885399	0.245795	0.369211	0.050*	
C19	0.1706 (2)	−0.1339 (2)	0.04553 (19)	0.0421 (5)	
C22	0.0582 (2)	0.13100 (19)	0.06521 (19)	0.0430 (5)	
C18	0.1568 (2)	−0.0974 (2)	0.1717 (2)	0.0465 (5)	
C21	0.1057 (2)	−0.08458 (19)	−0.03270 (18)	0.0403 (4)	
H21	0.147604	−0.119704	−0.109616	0.048*	
C4	0.7237 (2)	0.0973 (2)	0.2995 (2)	0.0486 (5)	
H4	0.778644	0.041646	0.248834	0.058*	
C20	0.2590 (2)	−0.2212 (2)	0.0089 (2)	0.0511 (5)	
C2	0.4933 (2)	0.1344 (2)	0.3766 (2)	0.0519 (6)	
H2	0.395996	0.106783	0.377810	0.062*	
C3	0.5770 (3)	0.0556 (2)	0.3007 (2)	0.0546 (6)	
H3	0.535511	−0.026734	0.249262	0.065*	
C13	0.2377 (3)	−0.1687 (2)	0.2053 (3)	0.0569 (6)	
C23	0.3070 (2)	0.0969 (2)	0.0516 (3)	0.0577 (6)	
H23A	0.327020	0.174278	0.049641	0.087*	
H23B	0.356563	0.038075	−0.007708	0.087*	
H23C	0.340683	0.108909	0.131721	0.087*	
C12	0.5754 (3)	0.5746 (2)	0.6888 (2)	0.0556 (6)	
H12A	0.565830	0.564851	0.760764	0.083*	
H12B	0.485780	0.592162	0.663120	0.083*	
H12C	0.653563	0.641771	0.706880	0.083*	
C17	0.0903 (3)	−0.0086 (3)	0.2608 (2)	0.0586 (6)	
H17	0.037260	0.040576	0.241326	0.070*	
C11	0.9833 (3)	0.4598 (3)	0.7088 (2)	0.0563 (6)	
H11A	1.078464	0.462640	0.745463	0.084*	
H11B	0.924690	0.380024	0.683006	0.084*	
H11C	0.937461	0.522671	0.767542	0.084*	
C16	0.1048 (3)	0.0049 (4)	0.3782 (3)	0.0756 (9)	
H16	0.061535	0.064059	0.438113	0.091*	
C24	0.3164 (3)	−0.2864 (3)	−0.1101 (3)	0.0691 (8)	
H24A	0.409581	−0.306274	−0.095669	0.104*	
H24B	0.327194	−0.233579	−0.147959	0.104*	
H24C	0.249199	−0.361111	−0.163052	0.104*	
C14	0.2508 (3)	−0.1543 (3)	0.3241 (3)	0.0760 (9)	
H14	0.304350	−0.202074	0.345346	0.091*	
C15	0.1829 (4)	−0.0684 (4)	0.4080 (3)	0.0858 (11)	
H15	0.189089	−0.058611	0.487213	0.103*	
H1	0.414 (4)	0.357 (3)	0.548 (3)	0.073 (9)*	
H3A	0.375 (4)	−0.280 (3)	0.111 (3)	0.095 (11)*	

### 1,4-Dimethyl-3,6-bis(2-methylindol-3-yl)piperazine-2,5-dione dimethyl sulfoxide monosolvate (II) Atomic displacement parameters (Å^2^)

**Table:**

	*U*^11^	*U*^22^	*U*^33^	*U*^12^	*U*^13^	*U*^23^
O1	0.0336 (8)	0.0773 (12)	0.0873 (12)	0.0219 (8)	0.0136 (8)	0.0533 (10)
O2	0.0573 (10)	0.0392 (9)	0.0720 (12)	0.0033 (7)	−0.0119 (9)	0.0077 (8)
N2	0.0262 (8)	0.0467 (9)	0.0451 (9)	0.0066 (7)	0.0039 (7)	0.0205 (8)
N4	0.0352 (9)	0.0410 (9)	0.0461 (10)	0.0036 (7)	0.0004 (7)	0.0165 (8)
N1	0.0245 (8)	0.0544 (11)	0.0559 (11)	0.0062 (7)	0.0117 (7)	0.0194 (9)
C6	0.0272 (9)	0.0402 (10)	0.0395 (10)	0.0052 (7)	0.0041 (7)	0.0185 (8)
C10	0.0227 (9)	0.0392 (10)	0.0544 (12)	0.0045 (7)	0.0017 (8)	0.0200 (9)
C7	0.0243 (9)	0.0418 (10)	0.0408 (10)	0.0060 (7)	0.0050 (7)	0.0153 (9)
N3	0.0533 (12)	0.0568 (13)	0.0852 (16)	0.0139 (10)	−0.0035 (11)	0.0370 (12)
C9	0.0247 (9)	0.0357 (10)	0.0441 (10)	0.0069 (7)	0.0048 (7)	0.0121 (8)
C1	0.0293 (9)	0.0456 (11)	0.0474 (11)	0.0055 (8)	0.0061 (8)	0.0212 (9)
C8	0.0287 (9)	0.0475 (11)	0.0436 (11)	0.0082 (8)	0.0068 (8)	0.0165 (9)
C5	0.0325 (10)	0.0434 (11)	0.0480 (12)	0.0080 (8)	0.0093 (8)	0.0189 (9)
C19	0.0359 (10)	0.0442 (11)	0.0437 (11)	0.0075 (8)	0.0044 (8)	0.0185 (9)
C22	0.0432 (11)	0.0401 (11)	0.0371 (10)	0.0048 (9)	−0.0028 (8)	0.0123 (9)
C18	0.0331 (10)	0.0582 (13)	0.0500 (12)	0.0017 (9)	0.0023 (9)	0.0289 (11)
C21	0.0404 (11)	0.0399 (11)	0.0342 (10)	0.0091 (8)	0.0059 (8)	0.0112 (8)
C4	0.0473 (12)	0.0426 (11)	0.0514 (13)	0.0120 (9)	0.0115 (10)	0.0166 (10)
C20	0.0419 (12)	0.0436 (12)	0.0617 (14)	0.0084 (9)	0.0021 (10)	0.0197 (11)
C2	0.0343 (11)	0.0515 (13)	0.0618 (14)	−0.0041 (9)	0.0031 (10)	0.0229 (11)
C3	0.0509 (13)	0.0411 (12)	0.0599 (14)	−0.0003 (10)	0.0036 (11)	0.0163 (11)
C13	0.0442 (12)	0.0642 (15)	0.0688 (16)	−0.0034 (11)	−0.0046 (11)	0.0415 (13)
C23	0.0367 (12)	0.0583 (14)	0.0704 (16)	0.0011 (10)	−0.0013 (11)	0.0266 (13)
C12	0.0387 (11)	0.0606 (14)	0.0535 (13)	0.0165 (10)	0.0133 (10)	0.0121 (11)
C17	0.0415 (12)	0.0866 (18)	0.0497 (13)	0.0120 (12)	0.0113 (10)	0.0327 (13)
C11	0.0424 (12)	0.0770 (17)	0.0587 (14)	0.0101 (11)	0.0062 (10)	0.0399 (13)
C16	0.0521 (15)	0.123 (3)	0.0509 (15)	0.0039 (16)	0.0127 (12)	0.0420 (17)
C24	0.0587 (15)	0.0563 (15)	0.0728 (18)	0.0226 (12)	0.0116 (13)	0.0102 (13)
C14	0.0594 (16)	0.101 (2)	0.087 (2)	−0.0105 (16)	−0.0146 (15)	0.071 (2)
C15	0.0646 (18)	0.140 (3)	0.0644 (18)	−0.014 (2)	0.0007 (15)	0.067 (2)

### 1,4-Dimethyl-3,6-bis(2-methylindol-3-yl)piperazine-2,5-dione dimethyl sulfoxide monosolvate (II) Geometric parameters (Å, º)

**Table:**

O1—C10	1.232 (3)	C18—C13	1.407 (3)
O2—C22	1.226 (3)	C18—C17	1.400 (4)
N2—C10	1.336 (3)	C21—H21	0.9800
N2—C9	1.471 (2)	C4—H4	0.9300
N2—C11	1.457 (3)	C4—C3	1.400 (3)
N4—C22	1.334 (3)	C20—C24	1.494 (4)
N4—C21	1.468 (3)	C2—H2	0.9300
N4—C23	1.465 (3)	C2—C3	1.374 (3)
N1—C1	1.373 (3)	C3—H3	0.9300
N1—C8	1.373 (3)	C13—C14	1.395 (4)
N1—H1	0.85 (3)	C23—H23A	0.9600
C6—C7	1.438 (3)	C23—H23B	0.9600
C6—C1	1.414 (3)	C23—H23C	0.9600
C6—C5	1.402 (3)	C12—H12A	0.9600
C10—C9^i^	1.517 (3)	C12—H12B	0.9600
C7—C9	1.506 (3)	C12—H12C	0.9600
C7—C8	1.371 (3)	C17—H17	0.9300
N3—C20	1.372 (3)	C17—C16	1.382 (4)
N3—C13	1.377 (4)	C11—H11A	0.9600
N3—H3A	0.92 (4)	C11—H11B	0.9600
C9—H9	0.9800	C11—H11C	0.9600
C1—C2	1.389 (3)	C16—H16	0.9300
C8—C12	1.488 (3)	C16—C15	1.387 (5)
C5—H5	0.9300	C24—H24A	0.9600
C5—C4	1.377 (3)	C24—H24B	0.9600
C19—C18	1.435 (3)	C24—H24C	0.9600
C19—C21	1.495 (3)	C14—H14	0.9300
C19—C20	1.368 (3)	C14—C15	1.361 (5)
C22—C21^ii^	1.520 (3)	C15—H15	0.9300
			
C10—N2—C9	125.04 (18)	C5—C4—H4	119.3
C10—N2—C11	119.54 (18)	C5—C4—C3	121.4 (2)
C11—N2—C9	114.98 (17)	C3—C4—H4	119.3
C22—N4—C21	124.73 (18)	N3—C20—C24	120.2 (2)
C22—N4—C23	118.72 (19)	C19—C20—N3	109.0 (2)
C23—N4—C21	115.63 (18)	C19—C20—C24	130.7 (2)
C1—N1—C8	109.87 (17)	C1—C2—H2	121.2
C1—N1—H1	128 (2)	C3—C2—C1	117.7 (2)
C8—N1—H1	121 (2)	C3—C2—H2	121.2
C1—C6—C7	106.30 (17)	C4—C3—H3	119.5
C5—C6—C7	135.52 (18)	C2—C3—C4	121.0 (2)
C5—C6—C1	118.17 (18)	C2—C3—H3	119.5
O1—C10—N2	121.9 (2)	N3—C13—C18	107.6 (2)
O1—C10—C9^i^	118.61 (18)	N3—C13—C14	130.6 (3)
N2—C10—C9^i^	119.49 (17)	C14—C13—C18	121.7 (3)
C6—C7—C9	126.94 (17)	N4—C23—H23A	109.5
C8—C7—C6	107.46 (17)	N4—C23—H23B	109.5
C8—C7—C9	125.59 (18)	N4—C23—H23C	109.5
C20—N3—C13	109.4 (2)	H23A—C23—H23B	109.5
C20—N3—H3A	125 (2)	H23A—C23—H23C	109.5
C13—N3—H3A	122 (2)	H23B—C23—H23C	109.5
N2—C9—C10^i^	114.68 (16)	C8—C12—H12A	109.5
N2—C9—C7	110.94 (16)	C8—C12—H12B	109.5
N2—C9—H9	107.0	C8—C12—H12C	109.5
C10^i^—C9—H9	107.0	H12A—C12—H12B	109.5
C7—C9—C10^i^	109.76 (16)	H12A—C12—H12C	109.5
C7—C9—H9	107.0	H12B—C12—H12C	109.5
N1—C1—C6	107.51 (18)	C18—C17—H17	120.6
N1—C1—C2	129.92 (19)	C16—C17—C18	118.9 (3)
C2—C1—C6	122.56 (19)	C16—C17—H17	120.6
N1—C8—C12	119.88 (18)	N2—C11—H11A	109.5
C7—C8—N1	108.82 (18)	N2—C11—H11B	109.5
C7—C8—C12	131.2 (2)	N2—C11—H11C	109.5
C6—C5—H5	120.4	H11A—C11—H11B	109.5
C4—C5—C6	119.18 (19)	H11A—C11—H11C	109.5
C4—C5—H5	120.4	H11B—C11—H11C	109.5
C18—C19—C21	127.36 (19)	C17—C16—H16	119.5
C20—C19—C18	107.4 (2)	C17—C16—C15	121.0 (3)
C20—C19—C21	125.2 (2)	C15—C16—H16	119.5
O2—C22—N4	122.9 (2)	C20—C24—H24A	109.5
O2—C22—C21^ii^	117.74 (19)	C20—C24—H24B	109.5
N4—C22—C21^ii^	119.35 (18)	C20—C24—H24C	109.5
C13—C18—C19	106.5 (2)	H24A—C24—H24B	109.5
C17—C18—C19	134.7 (2)	H24A—C24—H24C	109.5
C17—C18—C13	118.8 (2)	H24B—C24—H24C	109.5
N4—C21—C19	111.20 (17)	C13—C14—H14	121.0
N4—C21—C22^ii^	114.68 (17)	C15—C14—C13	118.0 (3)
N4—C21—H21	106.7	C15—C14—H14	121.0
C19—C21—C22^ii^	110.40 (17)	C16—C15—H15	119.2
C19—C21—H21	106.7	C14—C15—C16	121.6 (3)
C22^ii^—C21—H21	106.7	C14—C15—H15	119.2
			
N1—C1—C2—C3	−177.7 (2)	C22—N4—C21—C19	−113.3 (2)
C6—C7—C9—N2	−56.4 (3)	C22—N4—C21—C22^ii^	12.9 (3)
C6—C7—C9—C10^i^	71.4 (2)	C18—C19—C21—N4	55.7 (3)
C6—C7—C8—N1	−1.1 (2)	C18—C19—C21—C22^ii^	−72.7 (3)
C6—C7—C8—C12	176.2 (2)	C18—C19—C20—N3	2.1 (3)
C6—C1—C2—C3	1.6 (4)	C18—C19—C20—C24	−176.6 (2)
C6—C5—C4—C3	1.1 (3)	C18—C13—C14—C15	−0.1 (4)
C10—N2—C9—C10^i^	−10.2 (3)	C18—C17—C16—C15	−0.4 (4)
C10—N2—C9—C7	114.8 (2)	C21—N4—C22—O2	168.4 (2)
C7—C6—C1—N1	−1.8 (2)	C21—N4—C22—C21^ii^	−13.4 (3)
C7—C6—C1—C2	178.7 (2)	C21—C19—C18—C13	−179.5 (2)
C7—C6—C5—C4	179.5 (2)	C21—C19—C18—C17	−2.8 (4)
N3—C13—C14—C15	177.8 (3)	C21—C19—C20—N3	−179.7 (2)
C9—N2—C10—O1	−171.18 (19)	C21—C19—C20—C24	1.6 (4)
C9—N2—C10—C9^i^	10.7 (3)	C20—N3—C13—C18	1.3 (3)
C9—C7—C8—N1	178.31 (19)	C20—N3—C13—C14	−176.9 (3)
C9—C7—C8—C12	−4.3 (4)	C20—C19—C18—C13	−1.3 (2)
C1—N1—C8—C7	0.0 (3)	C20—C19—C18—C17	175.3 (3)
C1—N1—C8—C12	−177.7 (2)	C20—C19—C21—N4	−122.1 (2)
C1—C6—C7—C9	−177.62 (19)	C20—C19—C21—C22^ii^	109.4 (2)
C1—C6—C7—C8	1.8 (2)	C13—N3—C20—C19	−2.1 (3)
C1—C6—C5—C4	0.8 (3)	C13—N3—C20—C24	176.7 (2)
C1—C2—C3—C4	0.3 (4)	C13—C18—C17—C16	−0.8 (4)
C8—N1—C1—C6	1.2 (2)	C13—C14—C15—C16	−1.1 (4)
C8—N1—C1—C2	−179.4 (2)	C23—N4—C22—O2	−0.1 (3)
C8—C7—C9—N2	124.3 (2)	C23—N4—C22—C21^ii^	178.1 (2)
C8—C7—C9—C10^i^	−108.0 (2)	C23—N4—C21—C19	55.6 (2)
C5—C6—C7—C9	3.5 (4)	C23—N4—C21—C22^ii^	−178.29 (19)
C5—C6—C7—C8	−177.0 (2)	C17—C18—C13—N3	−177.2 (2)
C5—C6—C1—N1	177.29 (19)	C17—C18—C13—C14	1.1 (4)
C5—C6—C1—C2	−2.2 (3)	C17—C16—C15—C14	1.4 (5)
C5—C4—C3—C2	−1.6 (4)	C11—N2—C10—O1	0.8 (3)
C19—C18—C13—N3	0.0 (2)	C11—N2—C10—C9^i^	−177.35 (19)
C19—C18—C13—C14	178.4 (2)	C11—N2—C9—C10^i^	177.48 (18)
C19—C18—C17—C16	−177.1 (3)	C11—N2—C9—C7	−57.5 (2)

Symmetry codes: (i) −*x*+2, −*y*+1, −*z*+1; (ii) −*x*, −*y*, −*z*.

### 1,4-Dimethyl-3,6-bis(2-methylindol-3-yl)piperazine-2,5-dione dimethyl sulfoxide monosolvate (II) Hydrogen-bond geometry (Å, º)

**Table:**

*D*—H···*A*	*D*—H	H···*A*	*D*···*A*	*D*—H···*A*
N1—H1···O1^iii^	0.85 (3)	2.11 (3)	2.950 (2)	168 (3)

Symmetry code: (iii) *x*−1, *y*, *z*.

### 1,4-Dimethyl-3,6-bis(2-methylindol-3-yl)piperazine-2,5-dione tetrahydrofuran monosolvate (III) Crystal data

**Table:**

C_24_H_24_N_4_O_2_·C_4_H_8_O	*F*(000) = 504
*M**_r_* = 472.57	*D*_x_ = 1.186 Mg m^−^^3^
Monoclinic, *P*2_1_/*c*	Mo *K*α radiation, λ = 0.71073 Å
*a* = 8.5276 (9) Å	Cell parameters from 1124 reflections
*b* = 8.9357 (7) Å	θ = 2.4–21.3°
*c* = 17.4883 (13) Å	µ = 0.08 mm^−^^1^
β = 96.731 (8)°	*T* = 293 K
*V* = 1323.4 (2) Å^3^	Irregular, clear light red
*Z* = 2	0.4 × 0.4 × 0.3 mm

### 1,4-Dimethyl-3,6-bis(2-methylindol-3-yl)piperazine-2,5-dione tetrahydrofuran monosolvate (III) Data collection

**Table:**

XtaLAB Mini (ROW) diffractometer	2420 independent reflections
Radiation source: fine-focus sealed X-ray tube, Rigaku (Mo) X-ray Source	1519 reflections with *I* > 2σ(*I*)
Graphite monochromator	*R*_int_ = 0.031
ω scans	θ_max_ = 25.4°, θ_min_ = 2.4°
Absorption correction: multi-scan (CrysAlisPro; Rigaku OD, 2023)	*h* = −9→10
*T*_min_ = 0.968, *T*_max_ = 1.000	*k* = −10→9
6423 measured reflections	*l* = −21→20

### 1,4-Dimethyl-3,6-bis(2-methylindol-3-yl)piperazine-2,5-dione tetrahydrofuran monosolvate (III) Refinement

**Table:**

Refinement on *F*^2^	Hydrogen site location: mixed
Least-squares matrix: full	H atoms treated by a mixture of independent and constrained refinement
*R*[*F*^2^ > 2σ(*F*^2^)] = 0.053	*w* = 1/[σ^2^(*F*_o_^2^) + (0.0989*P*)^2^] where *P* = (*F*_o_^2^ + 2*F*_c_^2^)/3
*wR*(*F*^2^) = 0.178	(Δ/σ)_max_ < 0.001
*S* = 1.02	Δρ_max_ = 0.26 e Å^−^^3^
2420 reflections	Δρ_min_ = −0.15 e Å^−^^3^
143 parameters	Extinction correction: SHELXL-2018/3 (Sheldrick 2015b), Fc^*^=kFc[1+0.001xFc^2^λ^3^/sin(2θ)]^-1/4^
0 restraints	Extinction coefficient: 0.019 (4)
Primary atom site location: dual	

### 1,4-Dimethyl-3,6-bis(2-methylindol-3-yl)piperazine-2,5-dione tetrahydrofuran monosolvate (III) Special details

**Table:**

Geometry. All esds (except the esd in the dihedral angle between two l.s. planes) are estimated using the full covariance matrix. The cell esds are taken into account individually in the estimation of esds in distances, angles and torsion angles; correlations between esds in cell parameters are only used when they are defined by crystal symmetry. An approximate (isotropic) treatment of cell esds is used for estimating esds involving l.s. planes.

### 1,4-Dimethyl-3,6-bis(2-methylindol-3-yl)piperazine-2,5-dione tetrahydrofuran monosolvate (III) Fractional atomic coordinates and isotropic or equivalent isotropic displacement parameters (Å^2^)

**Table:**

	*x*	*y*	*z*	*U*_iso_*/*U*_eq_	
O1	0.6513 (2)	0.59725 (19)	0.63282 (8)	0.0738 (6)	
N1	0.5240 (2)	0.65140 (17)	0.51655 (9)	0.0552 (5)	
N2	0.6285 (3)	0.7814 (2)	0.27788 (12)	0.0705 (6)	
C1	0.5781 (3)	0.5565 (2)	0.57146 (10)	0.0493 (6)	
C4	0.7086 (3)	0.6138 (2)	0.36953 (11)	0.0516 (6)	
C2	0.4530 (3)	0.6105 (2)	0.43922 (10)	0.0499 (6)	
H2A	0.350921	0.661728	0.430019	0.060*	
C3	0.5519 (3)	0.6631 (2)	0.37871 (11)	0.0550 (6)	
C9	0.7526 (3)	0.6901 (2)	0.30495 (11)	0.0587 (6)	
C10	0.5065 (3)	0.7634 (3)	0.32147 (12)	0.0648 (7)	
C5	0.8164 (3)	0.5138 (3)	0.40716 (12)	0.0623 (6)	
H5	0.791463	0.462054	0.450270	0.075*	
C8	0.8974 (3)	0.6686 (3)	0.27802 (14)	0.0700 (7)	
H8	0.924270	0.720385	0.235309	0.084*	
C7	0.9994 (3)	0.5686 (3)	0.31634 (15)	0.0779 (8)	
H7	1.097093	0.551645	0.299209	0.094*	
C6	0.9599 (3)	0.4919 (3)	0.38035 (15)	0.0759 (7)	
H6	1.031507	0.424606	0.405495	0.091*	
C12	0.5442 (5)	0.8121 (3)	0.53101 (16)	0.0991 (11)	
H12A	0.465945	0.866466	0.498128	0.149*	
H12B	0.647594	0.842222	0.520525	0.149*	
H12C	0.532183	0.833018	0.583857	0.149*	
C11	0.3572 (4)	0.8489 (3)	0.30298 (16)	0.0923 (10)	
H11A	0.379818	0.954165	0.304557	0.138*	
H11B	0.286236	0.825603	0.340035	0.138*	
H11C	0.309282	0.822071	0.252427	0.138*	
H2	0.622 (3)	0.825 (3)	0.2308 (16)	0.092 (9)*	

### 1,4-Dimethyl-3,6-bis(2-methylindol-3-yl)piperazine-2,5-dione tetrahydrofuran monosolvate (III) Atomic displacement parameters (Å^2^)

**Table:**

	*U*^11^	*U*^22^	*U*^33^	*U*^12^	*U*^13^	*U*^23^
O1	0.0805 (12)	0.0974 (13)	0.0430 (8)	−0.0108 (10)	0.0060 (8)	−0.0230 (8)
N1	0.0905 (15)	0.0337 (9)	0.0431 (9)	−0.0042 (9)	0.0150 (9)	−0.0044 (7)
N2	0.0864 (16)	0.0763 (13)	0.0498 (11)	−0.0047 (12)	0.0129 (11)	0.0245 (10)
C1	0.0623 (14)	0.0549 (12)	0.0330 (10)	−0.0011 (10)	0.0157 (9)	−0.0048 (9)
C4	0.0645 (15)	0.0517 (12)	0.0394 (11)	−0.0034 (10)	0.0100 (10)	0.0024 (9)
C2	0.0643 (14)	0.0462 (12)	0.0406 (11)	0.0089 (10)	0.0117 (10)	0.0061 (9)
C3	0.0722 (16)	0.0516 (12)	0.0423 (11)	0.0043 (11)	0.0115 (10)	0.0119 (9)
C9	0.0748 (17)	0.0593 (13)	0.0430 (11)	−0.0088 (12)	0.0111 (11)	0.0041 (10)
C10	0.0811 (18)	0.0644 (14)	0.0491 (12)	0.0045 (13)	0.0083 (12)	0.0189 (10)
C5	0.0703 (17)	0.0625 (14)	0.0560 (12)	0.0033 (12)	0.0144 (12)	0.0089 (11)
C8	0.0795 (19)	0.0754 (16)	0.0594 (14)	−0.0175 (14)	0.0261 (13)	0.0032 (12)
C7	0.0702 (18)	0.0842 (18)	0.0833 (18)	−0.0055 (15)	0.0254 (15)	−0.0084 (15)
C6	0.0714 (19)	0.0778 (17)	0.0798 (16)	0.0058 (14)	0.0143 (14)	0.0063 (14)
C12	0.168 (3)	0.0423 (13)	0.093 (2)	−0.0147 (16)	0.038 (2)	−0.0148 (13)
C11	0.098 (2)	0.092 (2)	0.0856 (19)	0.0211 (17)	0.0080 (17)	0.0409 (16)

### 1,4-Dimethyl-3,6-bis(2-methylindol-3-yl)piperazine-2,5-dione tetrahydrofuran monosolvate (III) Geometric parameters (Å, º)

**Table:**

O1—C1	1.231 (2)	C10—C11	1.487 (4)
N1—C1	1.323 (3)	C5—H5	0.9300
N1—C2	1.462 (3)	C5—C6	1.375 (3)
N1—C12	1.465 (3)	C8—H8	0.9300
N2—C9	1.376 (3)	C8—C7	1.366 (4)
N2—C10	1.369 (3)	C7—H7	0.9300
N2—H2	0.91 (3)	C7—C6	1.387 (3)
C1—C2^i^	1.524 (3)	C6—H6	0.9300
C4—C3	1.434 (3)	C12—H12A	0.9600
C4—C9	1.408 (3)	C12—H12B	0.9600
C4—C5	1.391 (3)	C12—H12C	0.9600
C2—H2A	0.9800	C11—H11A	0.9600
C2—C3	1.504 (3)	C11—H11B	0.9600
C3—C10	1.365 (3)	C11—H11C	0.9600
C9—C8	1.386 (4)		
			
C1—N1—C2	125.65 (16)	C3—C10—C11	131.2 (2)
C1—N1—C12	118.62 (19)	C4—C5—H5	120.2
C2—N1—C12	115.62 (18)	C6—C5—C4	119.7 (2)
C9—N2—H2	122.0 (18)	C6—C5—H5	120.2
C10—N2—C9	110.06 (18)	C9—C8—H8	121.1
C10—N2—H2	125.8 (18)	C7—C8—C9	117.8 (2)
O1—C1—N1	122.78 (19)	C7—C8—H8	121.1
O1—C1—C2^i^	117.35 (19)	C8—C7—H7	119.4
N1—C1—C2^i^	119.85 (18)	C8—C7—C6	121.2 (2)
C9—C4—C3	106.31 (19)	C6—C7—H7	119.4
C5—C4—C3	135.82 (19)	C5—C6—C7	121.0 (3)
C5—C4—C9	117.9 (2)	C5—C6—H6	119.5
N1—C2—C1^i^	113.94 (15)	C7—C6—H6	119.5
N1—C2—H2A	107.4	N1—C12—H12A	109.5
N1—C2—C3	111.53 (18)	N1—C12—H12B	109.5
C1^i^—C2—H2A	107.4	N1—C12—H12C	109.5
C3—C2—C1^i^	108.90 (15)	H12A—C12—H12B	109.5
C3—C2—H2A	107.4	H12A—C12—H12C	109.5
C4—C3—C2	126.14 (17)	H12B—C12—H12C	109.5
C10—C3—C4	107.89 (19)	C10—C11—H11A	109.5
C10—C3—C2	126.0 (2)	C10—C11—H11B	109.5
N2—C9—C4	107.2 (2)	C10—C11—H11C	109.5
N2—C9—C8	130.2 (2)	H11A—C11—H11B	109.5
C8—C9—C4	122.5 (2)	H11A—C11—H11C	109.5
N2—C10—C11	120.36 (19)	H11B—C11—H11C	109.5
C3—C10—N2	108.5 (2)		
			
N1—C2—C3—C4	64.2 (3)	C9—N2—C10—C3	1.6 (3)
N1—C2—C3—C10	−117.7 (2)	C9—N2—C10—C11	−179.2 (2)
N2—C9—C8—C7	−179.5 (2)	C9—C4—C3—C2	178.71 (19)
C1—N1—C2—C1^i^	8.7 (3)	C9—C4—C3—C10	0.3 (2)
C1—N1—C2—C3	−115.1 (2)	C9—C4—C5—C6	−0.5 (3)
C1^i^—C2—C3—C4	−62.4 (3)	C9—C8—C7—C6	−0.4 (4)
C1^i^—C2—C3—C10	115.7 (2)	C10—N2—C9—C4	−1.4 (3)
C4—C3—C10—N2	−1.2 (3)	C10—N2—C9—C8	178.3 (2)
C4—C3—C10—C11	179.8 (3)	C5—C4—C3—C2	−0.4 (4)
C4—C9—C8—C7	0.2 (4)	C5—C4—C3—C10	−178.8 (2)
C4—C5—C6—C7	0.3 (4)	C5—C4—C9—N2	180.0 (2)
C2—N1—C1—O1	172.4 (2)	C5—C4—C9—C8	0.3 (3)
C2—N1—C1—C2^i^	−9.1 (4)	C8—C7—C6—C5	0.2 (4)
C2—C3—C10—N2	−179.6 (2)	C12—N1—C1—O1	−3.5 (3)
C2—C3—C10—C11	1.4 (4)	C12—N1—C1—C2^i^	174.9 (2)
C3—C4—C9—N2	0.7 (2)	C12—N1—C2—C1^i^	−175.3 (2)
C3—C4—C9—C8	−179.1 (2)	C12—N1—C2—C3	60.9 (3)
C3—C4—C5—C6	178.6 (2)		

Symmetry code: (i) −*x*+1, −*y*+1, −*z*+1.

### 1,4-Dimethyl-3,6-bis(2-methylindol-3-yl)piperazine-2,5-dione tetrahydrofuran monosolvate (III) Hydrogen-bond geometry (Å, º)

**Table:**

*D*—H···*A*	*D*—H	H···*A*	*D*···*A*	*D*—H···*A*
N2—H2···O1^ii^	0.91 (3)	1.89 (3)	2.787 (2)	168 (3)

Symmetry code: (ii) *x*, −*y*+3/2, *z*−1/2.
